# Survival Rate of Atraumatic Restorative Treatment Restorations in Primary Posterior Teeth in Children with High Risk of Caries in the Republic of Kosovo—1-Year Follow-up

**DOI:** 10.1055/s-0042-1757907

**Published:** 2022-12-13

**Authors:** Rina Prokshi, Elizabeta Gjorgievska, Brikena Prokshi, Mirlinda Sopi, Miranda Sejdiu

**Affiliations:** 1 Department of Pediatric and Preventive Dentistry, Dentistry School, University of Pristina, Pristina, Kosovo; 2 Department of Pediatric and Preventive Dentistry, Faculty of Dentistry, Ss. Cyril and Methodius University, Skopje, Republic of North Macedonia; 3 Department of Pharmacy, Faculty of Medicine, University of Pristina, Pristina, Kosovo; 4 Department of Periodontology and Oral Medicine, Dentistry School, University of Pristina, Pristina, Kosovo; 5 Department of Dentistry, Faculty of Medicine, University of Pristina, Pristina, Kosovo

**Keywords:** atraumatic restorative treatment, disadvantaged populations, glass-ionomer cement, primary teeth

## Abstract

**Objective** Atraumatic restorative treatment (ART) may be beneficial for disadvantaged populations with no or limited access to dental services. This study aimed to evaluate the survival rate of single-surface ART restorations in primary posterior teeth in children with high caries risk.

**Materials and Methods** This study was conducted in six rural areas of the Republic of Kosovo, and 100 children aged 3 to 8 years participated in the study. Information was obtained from each parent/guardian regarding their children, such as sociodemographic characteristics, general health, dental history, dietary habits, oral hygiene, and fluoride exposure. The reduced Cariogram was used to estimate the risk of caries in the participants based on the seven factors specified in the program, and all the provided information were collected and entered in a computer program of the Cariogram. A pediatric dentist, accompanied by two assistants, performed 100 ART restorations in school settings using high-viscosity glass-ionomer cement (Fuji IX) following the nine steps of the ART procedure. The restorations were evaluated at 3, 6, 9, and 12 months using the ART restoration criteria.

**Statistical Analysis** Percentages, mean value, standard deviation, mean interquartile range, and difference test between arithmetic mean values were used to analyze the research results.

**Results** Review of the average of reduced Cariogram showed that the majority of children, 72%, were at high risk of developing caries, with only 28% having a good chance of avoiding caries in the future. A total of 77% of the children in the study had never visited dentists before due to poor economic conditions and the lack of dentists in the area. The success rates of ART restorations performed in single-surface cavities in primary teeth were very encouraging, with more than 97% success rates after a 1-year follow-up period.

**Conclusion** Our results demonstrate that ART is efficient, affordable, and practical for the treatment of single-surface cavities in primary posterior teeth. Owing to its low price and atraumatic nature, ART can potentially help disadvantaged children in Kosovo access dental care.

## Introduction

Oral health is of great importance in determining overall health, well-being, and quality of life. 1 The Global Burden of Disease Study 2019 in their research estimated that oral diseases are widespread and affect close to 3.5 billion people worldwide; caries of permanent teeth are the most common condition. Global estimates are staggering; caries of permanent teeth are prevalent in 2 billion people, with more than 520 million children facing caries of primary teeth. 2 We can freely state that dental caries is a biofilm-mediated, diet modulated, multifactorial, noncommunicable, dynamic disease resulting in net mineral loss of dental hard tissues. 3 4

In low economic status countries, there is less investment in health care and prevention; therefore, people have limited access to oral health care, and teeth remain untreated for long periods of time, and often extraction is the main treatment method. 1 5 Dental caries left untreated can affect the patient on many levels and can cause functional, esthetic, and psychosocial disorders, especially in children and adolescents. Such untreated conditions can pose a serious threat to children's overall health, and there is a tremendous risk of developing other diseases and conditions, such as osteomyelitis, infections of the throat and floor of the mouth, and systemic sepsis. 6

Atraumatic restorative treatment (ART) can be beneficial for disadvantaged populations with little or no access to dental services. ART can be described as a substitutive, low-cost, restorative treatment that can be performed with basic dental equipment. 7 The treatment is beneficial not only because of the use of only hand instruments but also because the process is performed without anesthesia or any electrically driven equipment; the cavity is restored with adhesive restorative material, usually a high-viscosity glass-ionomer cement (HVGIC). 8

A recent systematic review reported that single-surface ART restorations in both primary and permanent teeth have excellent survival rates and, therefore, may be implemented in clinical practice. Multiple-surface ART restoration, on the other hand, has a relatively low survival rate. 9 No significant difference was reported by several authors that compared the survival percentage of ART and traditional (amalgam/resin composite) treatment in primary and permanent dentition in single- or multiple-surface cavities. 10 11

HVGIC (Fuji IX) has contributed to the success rate of ART restorations owing to its biological, physical, and chemical properties. In particular, the physical and chemical adhesion of HVGIC, as well as the release and uptake of fluoride, make this an excellent material for ART restorations. 12 13 No less important is the moisture tolerance of this material which makes it favorable for younger patients. 13 Nine steps of performing the ART restorations are described in the first ART manual, which is indicated by the roman numeral “IX” in Fuji IX. 14

ART is a great way of managing dental caries because it is a minimum intervention and minimally invasive approach and has strongly contributed to the development of minimal intervention dentistry (MID). 15 The main goal of MID is to preserve teeth health and keep them functional for a lifetime while minimizing the removal of healthy tooth structure. 16 MID consists of the following main aspects that should be applied in the patient's life: (1) early caries detection and caries risk assessment; (2) remineralization of demineralized tooth tissues; (3) optimal tooth decay preventive measures; (4) minimal interventions during cavity preparation; and (5) correction rather than substation of restorations. 8

Risk assessment of caries is an important component of the process of managing and preventing dental caries. The Cariogram, a computerized program used to assess caries risk and identify patients at high risk of caries, is displayed in a full as well as a reduced form. 17 18 In the full Cariogram, the patients are evaluated according to caries experience, plaque amount, diet, bacterial account, and saliva secretion, and results are shown as a pie or circle chart, representing the “the chance to avoid cavities” in the future. The sectors of the chart are as follows: “bacteria” (plaque amount and mutans streptococci amount), “diet” (lactobacilli amount and diet frequency), “susceptibility” (fluoride program, salivary secretion, and salivary buffering capacity), and “circumstances” (caries experience and medical history). 17 The reduced Cariogram, which eliminates bacterial and saliva testing, can be successfully used to identify caries risk in children, and is more recommended than the full Cariogram. 19 20

The objective of this study was to evaluate the survival rate of single-surface ART restorations in posterior primary teeth of children at high caries risk in the Republic of Kosovo.

## Materials and Methods

### Ethical Aspects

This longitudinal prospective study was approved by the Research Ethics Committee of the Faculty of Dentistry of Ss. Cyril and Methodius University in Skopje (N #02-264383) and the Research Ethics Committee of the Dental Chamber of Kosovo, Republic of Kosovo (N #07). The parents/guardians of every child who took part in the study were properly informed, and they provided a signed statement of consent.

### Study Design and Participant Selection

This study was conducted from September 2020 to December 2021 and explicitly focused on primary schools in the rural areas of the Republic of Kosovo. For the purpose of this study, we chose villages with significantly low economic and infrastructural development and distant locations where dental care for children was unavailable, such as Jezerc in Ferizaj, Shala e Bajgores in Mitrovica, Gradica and Vasileva in Drenica, and Pasome and Cecelia in Vushtrri. Because of poor living conditions and low quality of life, the population emigrated, and each village had only one school with a small number of children.

A total of 280 female and male children participated in the study, all of whom were aged 3 to 8 years. Preschoolers and primary school children (first, second, and third grades) were prepared, assessed, and examined in the comfort of their classroom, and received further instructions on oral health, paying special attention to oral hygiene/tooth brushing and sugar consumption. The following children were included: children whose parents/guardians signed the informed consent statement, children who agreed to participate, children with lack of access to oral health care, children of both sexes aged 3 to 8 years, collaborative children, children with good overall health, and children with high caries risk in primary teeth. Tooth inclusion criteria were caries lesions localized in the dentine class, approachability to hand instruments for ART procedure, lack of pain, lack of fistula or abscess, lack of pulp exposure, and lack of pathological mobility. Of 280 potential participants, 180 were rejected, 160 did not satisfy the inclusion criteria, and 20 refused to participate. The screening resulted in a final selection of 100 children with an average age of 6.0 ± 1.1 years, ranging from a minimum of 3 to a maximum of 8 years.

A questionnaire was prepared in accordance with the World Health Organization (WHO) 21 and the American Academy of Pediatric Dentistry 22 with modifications, including information such as sociodemographics, general health status, dental history, dietary habits, oral hygiene, and exposure to fluoride, which were collected from each parent/guardian regarding their child. Clinical examinations were performed using oral mirrors and standard explorers. Assessment of caries status was achieved using the decay, missing, filled teeth [dmft] index according to the WHO criteria, 20 and the Silness and Löe index 23 was used to assess the plaque level of the teeth. The reduced Cariogram was used to estimate caries risk in the participants based on the seven factors indicated in the program, 17 18 and all the provided information were collected and entered in a computer program of the Cariogram ( Table 1 ).

**Table 1:** Caries-related factors according to the reduced Cariogram 17 18

Factor	Explanation and data collection	Cariogram score
Caries experience	Past caries experience, several new cavities that definitely appeared during the preceding year should score “3,” regardless of whether the number of fillings is low; data from dmft index	0: caries free and no filling 1: better than normal 2: normal for that age group 3: worse than normal
Related general disease	a General disease or conditions related to decay, medications	0: no disease 1:disease/conditions, mild degree 2: severe degree, long-lasting
Diet, content	a Estimation of cariogenic food eating per day, mean for “normal days”	0: very low fermentable carbohydrate (≤ 3) b 1: low fermentable carbohydrate, “noncariogenic” diet (4–8) b 2: moderate fermentable carbohydrate content (9–16) b 3: high fermentable carbohydrate intake appropriate diet (≥17) b
Diet, frequency	a Estimation of the number of meals per day, on average for “normal days”	0: maximum 3 per d 1: maximum 5 per d 2: maximum 7 per d 3: more than 7 per d
Plaque amount	Clinical examination; data on oral hygiene according to the Silness and Löe plaque index	0: extremely good oral hygiene, PI < 0.4 1: good oral hygiene, PI = 0.4–1.0 2: less than good oral hygiene, PI = 1.1–2.0 3: poor oral hygiene, PI > 2.0
F program	a Assessment of the F supply in the oral cavity	0: maximum F program 1: additional F measures, infrequently 2: F toothpaste only 3: avoiding F
Clinical judgment	Examiners own clinical and private scores for the individual patient	Dentist's opinion, “clinical feeling;” an automatic default score of 1

Abbreviations: dmft, decay, missing, filled teeth; F, fluoride; PI, plaque index; SD, standard deviation.

a Data from interview or questionnaire results.

b Number of food items containing sugar.

Source: This table was adapted from Bratthall et al 17 and Petersson et al. 18

### Treatment Procedure of ART Restoration—Nine Steps

The following instruments and materials were used for the procedure of ART restoration—(1) ART instruments ( *SSWhite/Duflex, Rio de Janeiro, Brazil* ): ART opener, dental hatchet, excavator (small, medium, and large), and applier/carver; (2) consumable material: cotton wool rolls, cotton wool pellets, cup, petroleum jelly, articulation paper, measuring spoon, mixing pad, and spatula; (3) miscellaneous: examination gloves, mouth mask, water, soap and towel sheet of textile, operating light, operation bed/headrest extension, small suitcase containing all the necessary items needed to perform treatment, and basket; (4) filling material: HVGIC ( *GC Fuji IX _GP_ , Belgium, Europe* ) and dentine conditioner ( *GC Cavity Conditioner, Belgium, Europe* ).

The ART guidelines were followed while performing the restorative treatments. 24 An empty classroom was adapted for treatment administration, and four to five children were treated per day during school hours. One pediatric dentist, accompanied by two assistants, performed 100 ART restorations by following these nine steps: ( **I** ) A mattress was placed on a table on which the child lay flat on the back and remained in the supine position. The operator sat firmly on the stool and his back was in a straight position. The assistant was positioned on the left side of the right-handed operator. ( **II** ) The operating site was isolated using cotton wool rolls. ( **III)** The pits and fissures were cleaned of plaque and food debris. This was achieved using an explorer and wet cotton wool pellets. Dry pellets were used for drying the tooth. After this, examination of the cavity was performed with a mirror and the explorer. ( **IV)** At small cavity openings, the cavity entrance was enlarged using an ART opener. The cavity edges were removed at locations where the enamel was extremely thin or demineralized using dental hatches. ( **V)** Decomposed dentine from the enamel–dentine junction was removed using a small excavator. The soft, decomposed dentine from the floor of the cavity was removed using medium and large excavators. After removing the soft and decomposed dentine, the cavity was washed with wet cotton pellets and dried using dry pellets. ( **VI** ) A dentine conditioner ( *GC Cavity Conditioner* ) was used to condition the cavity, pits, and fissures. The conditioner was applied using a cotton pellet, left for 10 seconds, and, afterward, washed with water. The cavity washing process included cotton wool pellets soaked in water, and the cavity was isolated with a cotton roll and dried with dried cotton pellets. ( **VII** ) Mixing the HVGIC ( *GC Fuji IX _GP_* ) was performed following the manufacturer's instructions, according to which the standard ratio of powder to liquid was 3.6 g:1.0 g (one level measuring spoon of powder to one drop of liquid). ( **VIII** ) An applier instrument was used to place the mixed HVGIC into the cavity, and an excavator's round surface was utilized to properly insert the mixture deeper into the cavity. By minimally overfilling the cavity and adding some glass ionomer, optimal filling was achieved for all potential pits and fissures adjoining the cavity. A thumb was used to thinly spread a small amount of petroleum jelly over the top of the gloved index finger. Then, the high glass ionomer was firmly pressed with the index finger into cavity pits and fissures, and 20 seconds later, the finger was removed from the tooth. A carver instrument or medium excavator was used to remove excess material; 1 to 2 minutes were needed until the material felt hard, while the tooth was kept dry. ( **IX** ) The bite was checked by asking the patient to bite on articulating paper placed on the restoration surface from side to side, and correction was made by a carver. Finally, petroleum jelly was applied to the ART restoration, and cotton–wool rolls were removed from the mouth. The patient received further instructions and was advised not to eat and drink for at least 1 hour.

### Evaluation of ART Restoration

The ART restoration criteria were used to evaluate the restorations. 24 The time period for restoration assessment was 3, 6, 9, and 12 months. A 0.5-mm ball end of metal community periodontal index probe was used for performing the assessments. Restorations that survived were represented by codes 0 (present, satisfactory) and 1 (present, small defect of less than 0.5 mm at the margin of the cavity) according to the scoring system. 24

## Statistical Methods

**Fig. 1:**
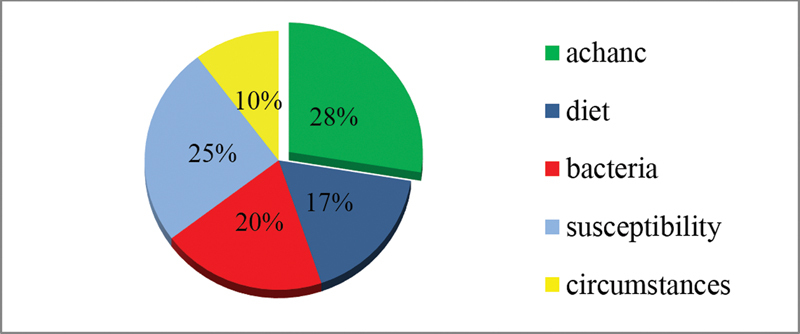
The average of reduced Cariogram for the entire group of children. Abbreviation: achanc, actual chance of avoiding new cavities.

All the data were processed using SPSS version 20.0. The research results were presented using the following statistical values: percentages, mean (average) value, standard deviation, mean interquartile range, and difference test between arithmetic mean values. These are presented in Tables 2 to 8 and Fig. 1 .

**Table 2:** Results of reduced Cariogram

Factor a	Cariogram score	Cariogram results *N* (%)
Caries experience	0–3	3: (100.0); mean dmft = 8.01 ± 2.3 (SD)
Related general disease	0–2	0: (98.0); 1: (2.0)
Diet, content	0–3	0: (2.0); 1: (55.0); 2: (41.0); 3: (2.0) b
Diet, frequency	0–3	1: (33.0); 2: (59.0); 3: (8.0) b
Plaque amount	0–3	1: (35.0); 2: (55.0); 3: (10.0) b ; mean PI = 1.3 ± 0.4 (SD)
Fluoride program	0–3	2: (92.0); 3: (8.0)
Clinical judgment	0–3	1: (100.0)

Abbreviations: dmft, decay, missing, filled teeth; PI, plaque index; SD, standard deviation.

a For each factor, the examiner must gather information by interviewing and examining the patient. The information is then rated on a scale from 0 to 3 (0–2 for some factors) according to predetermined criteria. The score “0” is the most favorable value, the maximum score “3” (or “2”) indicates high specified criteria and a high, unfavorable risk value.

b Statistically significant for *p* < 0.05.

**Table 3:** The average Cariogram for the entire group of children

Cariogram profile	Average	SD
ACHANC	27.7	11.77079
Diet	17.4	5.96881
Bacteria	20.0	4.11678
Susceptibility	25.1	10.05867
Circumstances	10.4	1.27837

Abbreviations: ACHANC, actual chance of avoiding new cavities; SD, standard deviation.

**Table 4:** Social demographic characteristics of children

	*N*	%
Sex a		
Female	56	56.0
Male	44	44.0
Education (father)		
Primary	54	54.0
High school	44	44.0
University	2	2.0
Education (mother)		
Primary	86	86.0
High school	14	14.0
Monthly income b		
< 100 euro	13	13.0
≤ 200 euro	63	63.0
200–400 euro	23	23.0
> 500 euro	1	1.0

a Statistically insignificant for *p* > 0.05 (difference test, *p* = .0897).

b Statistically significant for *p* < 0.05 (difference test, *p* = 0.0000).

**Table 5:** The frequency of tooth brushing

Brushing frequency a	*N*	% a
Once a day	38	38.0
Twice a day	21	21.0
More than twice a day	3	3.0
Several times a week	38	38.0

a Statistically different for *p* < 0.05 (difference test, *p* = 0.0084).

**Table 6:** The children's age on their first visit to a dentist

Age, y	*N*	%
0–1	2	2.0
1–3	6	6.0
3–6	7	7.0
6–8	8	8.0
Never	77	77.0

**Table 7:** Reason for lack of treatment

Reason for lack of treatment a	*N*	%
Lack of dentist	28	28.0
Low economic situation	55	55.0
Lack of time	13	13.0
Not deserving treatment	4	4.0

a Statistically significant for *p* < 0.05 (difference test, *p* = 0.0001).

**Table 8:** ART restoration score

Score/months of checkup	Present, satisfactory		Present, small defect at the margin of the cavity (less than 0.5 mm)	
	*N* r	%	*N*	%
3 mo	98	98.0	2	2.0
6 mo	98	98.0	2	2.0
9 mo	98	98.0	2	2.0
12 mo	97	97.0	3	3.0

Abbreviation: ART, atraumatic restorative treatment.

## Results

Only 3% of children in the current research brushed their teeth more than twice a day; 77% of the children in the study had never been to a dentist before due to low economic conditions and lack of dentists in the area. Of the mothers included in the research, 86% had only a primary education, and only 2% of the included fathers had a tertiary education, resulting in a condition where 99% of the participants lived with a family income of less than 500 euros.

The ART restoration score in children after 3, 6, and 9 months was not changed; it was satisfactory in 98 children, and a deficiency in the margin of 0.5 mm was noticed in only two children; after 12 months, the restoration was denoted as satisfactory in 97 children, while in 3 children, a deficiency of the margin of less than 0.5 mm was noticed. After 12 months, according to the index of the dynamics, a rate of decline of 1% was noticed with respect to the third, sixth, and ninth months.

## Discussion

This is the first study in the Republic of Kosovo to investigate the success rates of ART restorations in primary teeth in a school environment. Our findings corroborate earlier research findings that the ART technique is efficient, affordable, and practical for treating single-surface cavities in primary posterior teeth in less-developed regions.

Dental caries develops in primary teeth in a manner similar to that in permanent teeth. Primary teeth are significantly smaller than permanent teeth with thinner enamel and dentine layers. As a result, caries in primary teeth can progress considerably quicker into the dentine and afterward the pulp than in permanent teeth. Hence, caries in primary teeth must be prevented and managed because decay in primary teeth can be quite unpleasant and may cause a lot of pain, the experience of tooth removal can be frightening for a child, abscesses around the roots of primary teeth can damage the developing permanent teeth, and the early extraction of primary teeth may have a negative impact on the proper positioning of permanent teeth. 25

Some of the most crucial elements of primary health care are the prevention and proper treatment of common oral diseases. Low-income populations are especially at risk due to a variety of factors, including the absence of availability of dental care, the high cost of dental services, and a fundamental lack of knowledge about the significant role that oral health plays in general health and well-being. 7 26 Kosovo is a low-income country, which results in poor dental care habits; many young people do not visit dental clinics for preventive care or restorative treatment in order to keep their teeth healthy. Oral hygiene is generally inadequate, and oral health awareness and knowledge are often lacking.

One of the inclusion criteria in this study was a high caries risk. Overview of average of the reduced Cariogram showed that majority of children (72%) were at high risk for caries development, with only 28% having a good chance of avoiding caries in the future. The dominant sector in the risk profile in this study was susceptibility (25%), followed by bacteria (20%), diet (17%), and circumstances (10%). The caries profiles of children with primary dentition vary significantly in different regions. Cariogram of 5-year-old children in India showed that 66.2% of subjects had been assigned to high-risk category, where susceptibility (26%) was a predominant sector. 27 In opposition, a study conducted in Brazil showed that 8% of children aged 5 to 7 years were categorized into the high-risk category. 28

The mean dmft index in our study (average age, 6 years) was 8.01. In the study conducted by Begzati et al, 29 the mean dmft indices in 5- and 6-year-old children from Kosovo were 8.1 and 7.9, respectively. The mean dmft index of less-developed regions such as Albania (8.5), Bosnia (7.53), and Belarus (7.4) are in line with those in our study. 30

ART, as a component of a basic package of oral care, is a possible solution for preventing dental caries and arresting further progression in disadvantaged children in the Republic of Kosovo.

The results of our study showed that the success rates of ART restorations performed in single-surface cavities in primary teeth were highly promising, with more than 97% success rates after a 12-month follow-up period. This is in line with the findings of a study conducted in India. 31 The reported success rates for ART restorations vary between studies conducted in different countries. In comparison to the findings collected in our study for 1-year follow-up, Kuwait, 32 Turkey, 33 and Argentina 34 reported higher survival rates of single-surface ART restorations performed with HVGIC in primary posterior teeth. Although the survival rates in Iraq, Thailand, and Brazil were lower than ours, they were recorded to be 74, 35 79, 36 and 82%, 37 respectively.

Regardless of the remarkable survival rates in our study, the conclusions must be interpreted with caution, as only single-surface restorations were performed, and all ART restorations were carried out by pediatric dentists in school settings using HVGIC with a conditioner.

The causes of ART restoration success have been found to be multifactorial and include sufficient removal of demineralized enamel and soft decomposed dentine, proper mixing of glass-ionomer powder/liquid, degree of humidity and temperature when mixing glass ionomer, complete filling of the cavity with mixing glass ionomer, moisture control at the time of cavity filling, conditioning of the prepared cavity, and level of collaboration of the child. 38

Another factor associated with the success rate of ART restorations is the restoration type (single-surface vs. multiple-surface restoration). Multiple-surface ART restorations in primary teeth have been proven to have a lower survival rate than single-surface restorations. 36 39

Operators have a substantial impact on the success percentage of ART restorations in children. Jiang et al 40 reported that ART restorations placed by dental students/therapists had significantly lower success rates. Other variables, such as the environment (clinic or field) and the moisture control method (cotton roll, saliva ejector, or rubber dam) had no effect on the success rate of ART restorations. 40 Roshan and Sakeenabi 41 evaluated the success rate of ART restorations placed in a school setting and a clinical dental setup and concluded that there was no statistically significant difference between the ART restorations in both assessments.

Although the success rate of this study was high, some limitations should be considered. First, the sample size was small due to poor living conditions and quality of life; the population emigrated, resulting in a low number of school children. Another factor contributing to the small sample size was the difficulty in finding children who met the inclusion criteria. The second reservation about this study is that blinded evaluations could not be performed because only one qualified pediatric dentist conducted the ART restoration. Based on this aspect, operator training is essential for further investigations of the ART approach.

Due to its low price and atraumatic nature, ART may help Kosovo's disadvantaged children gain access to dental treatment. Furthermore, ART helps slow the spread of caries, improve dental health, reduce expenses, and save lives. In view of the fact that the Republic of Kosovo lacks a well-organized system for the prevention of oral diseases, ART should be incorporated into oral health programs in schools to support educational and preventive initiatives.

In this study, all participants received precise dietary and oral health instructions adapted to their ages. Brushing teeth twice a day with 1.000 to 1.500 ppm fluoride toothpaste was recommended for children aged 6 years, and 500 ppm fluoride toothpaste for younger children.

## Conclusion

The 1-year survival rate assessment of single-surface ART restorations in primary teeth was satisfactory. The ART approach was shown to have an effect on managing dental caries in children with high caries in the Republic of Kosovo.

## References

[1] World Health Organization Regional Office for Europe Oral HealthAccessed September 21, 2021, :https://www.euro.who.int/en/health-topics/disease-prevention/oral-health

[2] Global Burden of Disease Collaborative Network Global Burden of Disease Study 2019 (GBD 2019)Seattle: Institute of Health Metrics and Evaluation (IHME); 2020. Accessed January 30, 2022, at:https://vizhub.healthdata.org/gbd-results/

[3] Pitts N B Zero D T Marsh P D Dental caries Nat Rev Dis Primers 2017 3 17030 28540937 10.1038/nrdp.2017.30

[4] Machiulskiene V Campus G Carvalho J C Terminology of dental caries and dental caries management: consensus report of a workshop organized by ORCA and Cariology Research Group of IADR Caries Res 2020 54 01 7 14 31590168 10.1159/000503309

[5] Birant S Koruyucu M Ozcan H Investigating the level of knowledge of the community about oral and dental health Eur J Dent 2021 15 01 145 151 32932530 10.1055/s-0040-1716583 PMC7902119

[6] Peres M A Macpherson L MD Weyant R J Oral diseases: a global public health challenge Lancet 2019 394(10194)249 260 31327369 10.1016/S0140-6736(19)31146-8

[7] Estupiñán-Day S Milner T Tellez M Oral Health of Low-Income Children: Procedures for Atraumatic Restorative Treatment (PRAT) Washington, DC PAHO 2006

[8] Tyas M J Anusavice K J Frencken J E Mount G J Minimal intervention dentistry–a review. FDI Commission Project 1-97 Int Dent J 2000 50 01 1 12 10945174 10.1111/j.1875-595x.2000.tb00540.x

[9] de Amorim R G Frencken J E Raggio D P Chen X Hu X Leal S C Survival percentages of atraumatic restorative treatment (ART) restorations and sealants in posterior teeth: an updated systematic review and meta-analysis Clin Oral Investig 2018 22 08 2703 2725 10.1007/s00784-018-2625-5 30232622

[10] Mickenautsch S Yengopal V Banerjee A Atraumatic restorative treatment versus amalgam restoration longevity: a systematic review Clin Oral Investig 2010 14 03 233 240 10.1007/s00784-009-0335-8 19688227

[11] Tedesco T K Calvo A F Lenzi T L ART is an alternative for restoring occlusoproximal cavities in primary teeth - evidence from an updated systematic review and meta-analysis Int J Paediatr Dent 2017 27 03 201 209 27489205 10.1111/ipd.12252

[12] Frencken J E Adhesive restorative materials and ART London, United Kingdom Stephen Hancocks 2018 63 87

[13] Almuhaiza M Glass-ionomer cements in restorative dentistry: a critical appraisal J Contemp Dent Pract 2016 17 04 331 336 27340169 10.5005/jp-journals-10024-1850

[14] Frencken J E The evolution of ART London, United Kingdom Stephen Hancocks 2018 95

[15] Holmgren C J Roux D Doméjean S Minimal intervention dentistry: part 5. Atraumatic restorative treatment (ART)–a minimum intervention and minimally invasive approach for the management of dental caries Br Dent J 2013 214 01 11 18 23306489 10.1038/sj.bdj.2012.1175

[16] Frencken J E Peters M C Manton D J Leal S C Gordan V V Eden E Minimal intervention dentistry for managing dental caries - a review: report of a FDI task group Int Dent J 2012 62 05 223 243 23106836 10.1111/idj.12007 PMC3490231

[17] Bratthall D Petersson H Stjernswärd J R CARIOGRAM MANUAL a new and interactive way of illustrating the interaction of factors contributing to the development of dental cariesAccessed December 21, 2021, at:https://www.mah.se/upload/FAKULTETER/OD/cariogram%20program%20caries/cariogmanual201net.pdf

[18] Petersson G H Isberg P E Twetman S Caries risk assessment in school children using a reduced Cariogram model without saliva tests BMC Oral Health 2010 10 5 20403163 10.1186/1472-6831-10-5 PMC2864191

[19] Su N Lagerweij M D van der Heijden G JMG Assessment of predictive performance of caries risk assessment models based on a systematic review and meta-analysis J Dent 2021 110 103664 33984413 10.1016/j.jdent.2021.103664

[20] Taqi M Razak I A Ab-Murat N Caries risk assessment in school children using reduced Cariogram model Pak J Med Sci 2017 33 04 948 952 29067071 10.12669/pjms.334.13106 PMC5648970

[21] World Health Organization Petersen P E Baez R J Oral Health Surveys: Basic Methods 5th ed. Geneva World Health Organization 2013

[22] American Academy of Pediatric Dentirsty (AAPD) Pediatric Medical HistoryAccessed February 9, 2019, at:https://www.aapd.org/globalassets/media/policies_guidelines/r_medhistoryform.pdf

[23] Malmö University Oral Health Country/Area Profile Project. Methods and IndicesAccessed October 10, 2019, at:https://capp.mau.se/methods-and-indices

[24] Frencken J E ART restorations London, United Kingdom Stephen Hancocks 2018 149 155

[25] Frencken J E Manual for the Atraumatic Restorative Treatment Approach to Control Dental Caries 3rd ed. Groningen, The Netherlands WHO Collaborating Centre for Oral Health Services Research 1997

[26] Alshahrani N F Alshahrani A NA Alahmari M A Almanie A M Alosbi A M Togoo R A First dental visit: age, reason, and experiences of Saudi children Eur J Dent 2018 12 04 579 584 30369806 10.4103/ejd.ejd_426_17 PMC6178666

[27] Garg A Madan M Dua P Validating the usage of Cariogram in 5- and 12-year-old school-going children in Paonta Sahib, Himachal Pradesh, India: a 12-month prospective study Int J Clin Pediatr Dent 2018 11 02 110 115 29991863 10.5005/jp-journals-10005-1495 PMC6034047

[28] Cabral R N Hilgert L A Faber J Leal S C Caries risk assessment in schoolchildren–a form based on Cariogram software J Appl Oral Sci 2014 22 05 397 402 25466473 10.1590/1678-775720130689 PMC4245751

[29] Begzati A Meqa K Siegenthaler D Berisha M Mautsch W Dental health evaluation of children in Kosovo Eur J Dent 2011 5 01 32 39 21228954 PMC3019749

[30] Marthaler T M O'Mullane D M Vrbic V The prevalence of dental caries in Europe 1990-1995. ORCA Saturday afternoon symposium 1995 Caries Res 1996 30 04 237 255 8773416 10.1159/000262332

[31] Deepa G Shobha T A clinical evaluation of two glass ionomer cements in primary molars using atraumatic restorative treatment technique in India: 1 year follow up Int J Paediatr Dent 2010 20 06 410 418 20642467 10.1111/j.1365-263X.2010.01067.x

[32] Honkala E Behbehani J Ibricevic H Kerosuo E Al-Jame G The atraumatic restorative treatment (ART) approach to restoring primary teeth in a standard dental clinic Int J Paediatr Dent 2003 13 03 172 179 12752916 10.1046/j.1365-263x.2003.00455.x

[33] Ersin N K Candan U Aykut A Onçağ O Eronat C Kose T A clinical evaluation of resin-based composite and glass ionomer cement restorations placed in primary teeth using the ART approach: results at 24 months J Am Dent Assoc 2006 137 11 1529 1536 17082278 10.14219/jada.archive.2006.0087

[34] Molina G F Faulks D Mazzola I Cabral R J Mulder J Frencken J E Three-year survival of ART high-viscosity glass-ionomer and resin composite restorations in people with disability Clin Oral Investig 2018 22 01 461 467 10.1007/s00784-017-2134-y 28547182

[35] Yassen G One-year survival of occlusal ART restorations in primary molars placed with and without cavity conditioner J Dent Child (Chic) 2009 76 02 136 141 19619427

[36] Frencken J E Songpaisan Y Phantumvanit P Pilot T An atraumatic restorative treatment (ART) technique: evaluation after one year Int Dent J 1994 44 05 460 464 7814116

[37] Menezes J P Rosenblatt A Medeiros E Clinical evaluation of atraumatic restorations in primary molars: a comparison between 2 glass ionomer cements J Dent Child (Chic) 2006 73 02 91 97 16948370

[38] Frencken J E ART restorations London, United Kingdom Stephen Hancocks 2018 166

[39] da Franca C Colares V Van Amerongen E Two-year evaluation of the atraumatic restorative treatment approach in primary molars class I and II restorations Int J Paediatr Dent 2011 21 04 249 253 21401749 10.1111/j.1365-263X.2011.01125.x

[40] Jiang M Fan Y Li K Y Lo E CM Chu C H Wong M CM Factors affecting success rate of atraumatic restorative treatment (ART) restorations in children: a systematic review and meta-analysis J Dent 2021 104 103526 33188846 10.1016/j.jdent.2020.103526

[41] Roshan N M Sakeenabi B Survival of occlusal ART restorations in primary molars placed in school environment and hospital dental setup-one year follow-up study Med Oral Patol Oral Cir Bucal 2011 16 07 e973 e977 21743396 10.4317/medoral.17327

